# Nanostructured Valsartan Microparticles with Enhanced Bioavailability Produced by High-Throughput Electrohydrodynamic Room-Temperature Atomization

**DOI:** 10.1021/acs.molpharmaceut.1c00098

**Published:** 2021-06-28

**Authors:** Cristina Prieto, Zoran Evtoski, María Pardo-Figuerez, Julia Hrakovsky, Jose M. Lagaron

**Affiliations:** † Novel Materials and Nanotechnology Group, Institute of Agrochemistry and Food Technology (IATA), Spanish Council for Scientific Research (CSIC), Calle Catedrático Agustín Escardino Benlloch 7, 46980 Paterna, Valencia, Spain; ‡ Bioinicia R&D Department, Bioinicia S.L., Calle Algepser 65 nave 3, 46980 Paterna, Valencia, Spain; § R&D Finished Dosage Forms, Zakłady Farmaceutyczne Polpharma SA, ul. Pelplińska 19, 83-200 Starogard Gdański, Poland

**Keywords:** BCS II drug, valsartan, nanonization, HPMC, lactose monohydrate, enhanced bioavailability

## Abstract

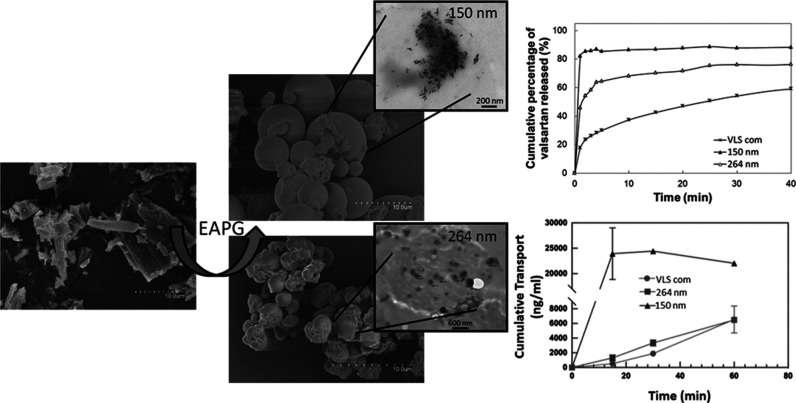

The high-throughput drying and encapsulation technique called electrospraying assisted by pressurized gas (EAPG) was used for the first time to produce nanostructured valsartan within microparticles of excipients. Valsartan, a poorly absorbed and lipid-soluble drug, was selected since it is considered a good model for BCS class II drugs. Two different polymeric matrices were selected as excipients, i.e., hydroxypropyl methylcellulose (HPMC) and lactose monohydrate, while Span 20 was used as a surfactant. The produced 80% valsartan loading formulations were characterized in terms of morphology, crystallinity, *in vitro* release, *in vitro* Caco-2 cells’ permeability, and *in vivo* pharmacokinetic study. Spherical microparticles of *ca*. 4 μm were obtained within which valsartan nanoparticles were seen to range from 150 to 650 nm. Wide-angle X-ray scattering and differential scanning calorimetry confirmed that valsartan had a lower and/or more ill-defined crystallinity than the commercial source, and photon correlation spectroscopy and transmission electron microscopy proved that it was dispersed and distributed in the form of nanoparticles of controlled size. *In vitro* dissolution tests showed that the HPMC formulation with the lowest API particle size, i.e., 150 nm, dissolved 2.5-fold faster than the commercial valsartan in the first 10 min. This formulation also showed a 4-fold faster *in vitro* permeability than the commercial valsartan and a 3-fold higher systemic exposure than the commercial sample. The results proved the potential of the EAPG processing technique for the production of safe-to-handle microparticles containing high quantities of a highly dispersed and distributed nanonized BCS class II model drug with enhanced bioavailability.

## 1 Introduction

Oral administration represents the most natural way for the intake of active pharmaceutical ingredients (APIs). However, oral drug delivery still has some major limitations related to the API chemical structure, its insufficient absorption across the gastrointestinal tract, and the considerable first pass effect, which can lead to a low bioavailability and pharmacokinetic behavior, causing a low therapeutic activity.^1,2^

Valsartan is a potent antihypertensive drug, which acts as a highly selective antagonist of the angiotensin II receptor type I (AT1 receptor subtype).^3−7^ According to the Biopharmaceutical Classification System (BCS), valsartan is a water-insoluble (3.08 μg/mL), lipophilic, and highly permeable class II compound, resulting in a low oral bioavailability (23%),^4,7,8^ a peak plasma concentration between 2 and 4 h after oral administration, and a plasma half-life of 7.5 h.^9^ Accordingly, new alternatives were evaluated to achieve the best therapeutic efficacy, such as cyclodextrin complexes, nanoparticles, solid dispersions, micro- and nanonization, self-emulsifying drug delivery systems, proniosomes, proliposomes, and mucoadhesive microspheres.^10−15^

Solid dispersions are considered one of the most effective approaches to improve drug solubility. They constitute an effective delivery system where drug molecules are dispersed in a carrier, either in molecular, amorphous particulate form or microcrystalline particulate form.^16,17^ The increased solubility is due to the high wettability, high porosity, reduced agglomeration and particle size, and the lack of lattice energy barrier to dissolution, which result in an enhanced bioavailability of the poorly water-soluble drugs.^18,19^ Some conventional methods such as melting, solvent evaporation, and solvent wetting were previously reported for the preparation of solid dispersions. However, these delivery systems present several disadvantages that reduce their applicability and efficiency, such as the use of high temperatures, which might chemically decompose the drug, the lack of control of the drug particle size, the low drug loading efficiencies, the toxicology of the large amounts of carriers required to improve the solubility of poorly water-soluble drugs, and the thermodynamic instability.^20^

Electrohydrodynamic processing, comprising electrospinning and electrospraying, consists of subjecting a polymer solution to a high electric field to generate ultrafine structures, which after solvent evaporation lead to nano- or microsized fibers or particles. The manufacturing process is carried out at room temperature, which avoids bioactive degradation, and without the need for further separation steps of the nano- or microstructures.^21^ The high electric field induces extensional forces to the polymeric higher-molecular-weight molecules, which are thought to more efficiently entrap the lower molecular weight molecules.^22−25^ The control of the particle-size distribution is produced by controlling the solution and process parameters such as voltage, which, in turn, finely break up the droplets *via* charged solvent molecules’ repulsion to a more homogeneous and defined particle size.^21^ Moreover, when the encapsulating polymer is conductive, it is attracted to the surface, thus promoting efficient encapsulation. Furthermore, the rapid solvent evaporation in the atomized droplets results in an amorphous state of the active ingredient,^26^ and together with the large variety of polymeric wall materials that can be used^27^ are the great advantages of this technique. Nevertheless, the low productivity of the process is the main drawback of this technology, which has hindered the scale-up for the industrial application.^28^

To tackle this limitation, Lagaron et al. developed an innovative room-temperature drying technique based on the combination of electrospraying with the pneumatic atomization process.^29^ This emerging high-throughput technology, termed electrospraying assisted by pressurized gas (EAPG), is based on the nebulization of a polymer solution by a pneumatic injector, the droplets of which are further exposed to a high electric field. The solvent in the nebulized droplets is evaporated at room temperature by virtue of the high voltage applied, and the dried material is then collected as a free-flowing powder. This technology was proven for the encapsulation of omega-3 rich oils in different matrices.^28−31^ In light of the appropriate selection of solution and process parameters, this technology allows producing nanosized materials dispersed and distributed within microparticles of a polymeric excipient, resulting in nanostructured amorphous API morphologies with controlled particle size and size distribution, hence preventing agglomeration. The characteristics of these new pharmaceutical forms could address the challenges of the solid dispersions and the nanoparticle technology. During the last years, the production of diverse pharmaceutical formulations using different therapeutic molecules in the form of particles or fibers by means of electrospinning and electrospraying techniques has been studied in depth.^32−36^ However, to the best of our knowledge, the production of nano-within-micro valsartan by electrospraying or EAPG has never been studied. However, Bukhary et al. did consider the use of the electrospinning technology to generate fast-dissolving valsartan and amlodipine besylate pharmaceutical formulations by coencapsulating both drugs in poly(vinylpirrolidone) fibers.^37^

In view of this, the aim of this seminal study was to evaluate the innovative nanostructuration of valsartan within excipient microparticles, which provides safe handling and prevents API aggregation, produced by the EAPG technology using hydroxypropyl methylcellulose (HPMC) and lactose monohydrate excipients. HPMC and lactose monohydrate excipients were selected because their solubility in water would enable an instant release of the API nanoparticles into solution after administration. Lactose monohydrate has instant solubility in water, whereas HPMC exhibits a somewhat slower solubilization rate. Morphological characterization of the microparticles was performed by scanning electron microscopy (SEM), transmission electron microscopy (TEM), and photon correlation spectroscopy. Potential changes in molecular order and/or stability of valsartan were ascertained by differential scanning calorimetry (DSC), wide-angle X-ray scattering (WAXS), and attenuated total reflection-Fourier transform infrared spectroscopy (ATR-FTIR). In addition, the *in vitro* release behavior of valsartan from the water-soluble microparticles was also characterized. Finally, determination of drug permeability and prediction of *in vitro* drug absorption with Caco-2 cell monolayers and *in vivo* pharmacokinetic study were also performed.

## 2 Materials and Methods

Valsartan was supplied by Polpharma (Starogard, Gdański, Poland). Hydroxypropyl methylcellulose (HPMC) from Lotte Fine Chemicals (Nam-gu, Ulsan, South Korea) and lactose monohydrate from DFE Pharma (Goch, Germany) were also supplied by Polpharma. Span 20 was acquired from Croda (Snaith, United Kingdom). Ethanol 96% (Ph. Eur.) was purchased from Panreac (Castellar del Vallès, Spain). Caco-2 cell line, Eagle’s minimum essential medium (EMEM), fetal bovine serum (FBS), Hank’s balanced salt solution (HBSS), and trypsin–ethylenediaminetetraacetic acid (EDTA) solution were purchased from ATCC (Manassas, VA). Penicillin–streptomycin, amphotericin B, phosphate-buffered saline (PBS), hydroclorothiazide, acetonitrile, sodium chloride, potassium chloride, sodium phosphate dibasic dihydrate, and potassium phosphate monobasic were purchased from Sigma-Aldrich (St. Louis, MO). A *N*-(2-hydroxyethyl)piperazine-*N*′-ethanesulfonic acid (HEPES) solution was purchased from Biowest (Nuaillé, France) and Corning Transwell polycarbonate membrane cell culture inserts were purchased from Corning (Corning, NY). Size 9 gelatin capsules (8.4 mm × 2.7 mm) were purchased from Torpac (Fairfield, NJ). Polyurethane #9 capsule dosers were purchased from Instech (Plymouth Meeting, PA). Deionized water was used throughout the study.

### 2.2 Solution Preparation

Four formulations of size-controlled valsartan were prepared in HPMC and lactose monohydrate, with or without Span 20. Emulsions were generated to control the particle size of valsartan within HPMC and lactose monohydrate microparticles. Different emulsion formulations were initially considered, but the selected ones provided good stability and different API sizes to compare the size effect in the study. The composition of each of the selected formulations is shown in Table 1. The organic and aqueous phases of the emulsions were prepared separately. In all formulations, valsartan was dissolved in an aqueous solution of ethanol, containing ethanol at 85% (v/v). The aqueous phase consisted of an aqueous solution of the polymers in water, with or without surfactant. The 30% organic phase was added carefully to the 70% aqueous phase and mixed using an Ultra-Turrax T25 (IKA-Werke GmbH, Staufen, Germany) at 11 000 rpm and room temperature. In all formulations, the valsartan loading in the microparticle was over 80%. These formulations were compared with the drug processed alone by the EAPG technology (VLS) and the commercial valsartan.

**Table 1:** Composition of the Valsartan Formulations Processed by the EAPG Technology

**formulation**	**aqueous phase**	**organic phase**	**drug/polymer/surfactant ratio**
VLS		120 mg/mL VLS in ethanol 85%	100:0:0
VEHS	10 and 1 mg/mL Span 20 HPMC in water	120 mg/mL VLS in ethanol 85%	82:16:2
VEH	10 mg/mL HPMC in water	120 mg/mL VLS in ethanol 85%	84:16:0
VELS	10 mg/mL lactose monohydrate and 0.8 mg/mL Span 20 in water	110 mg/mL VLS in ethanol 85%	82:17:1
VEL	10 mg/mL lactose monohydrate in water	110 mg/mL VLS in ethanol 85%	83:17:0

### 2.3 EAPG Process

**Figure 1:**
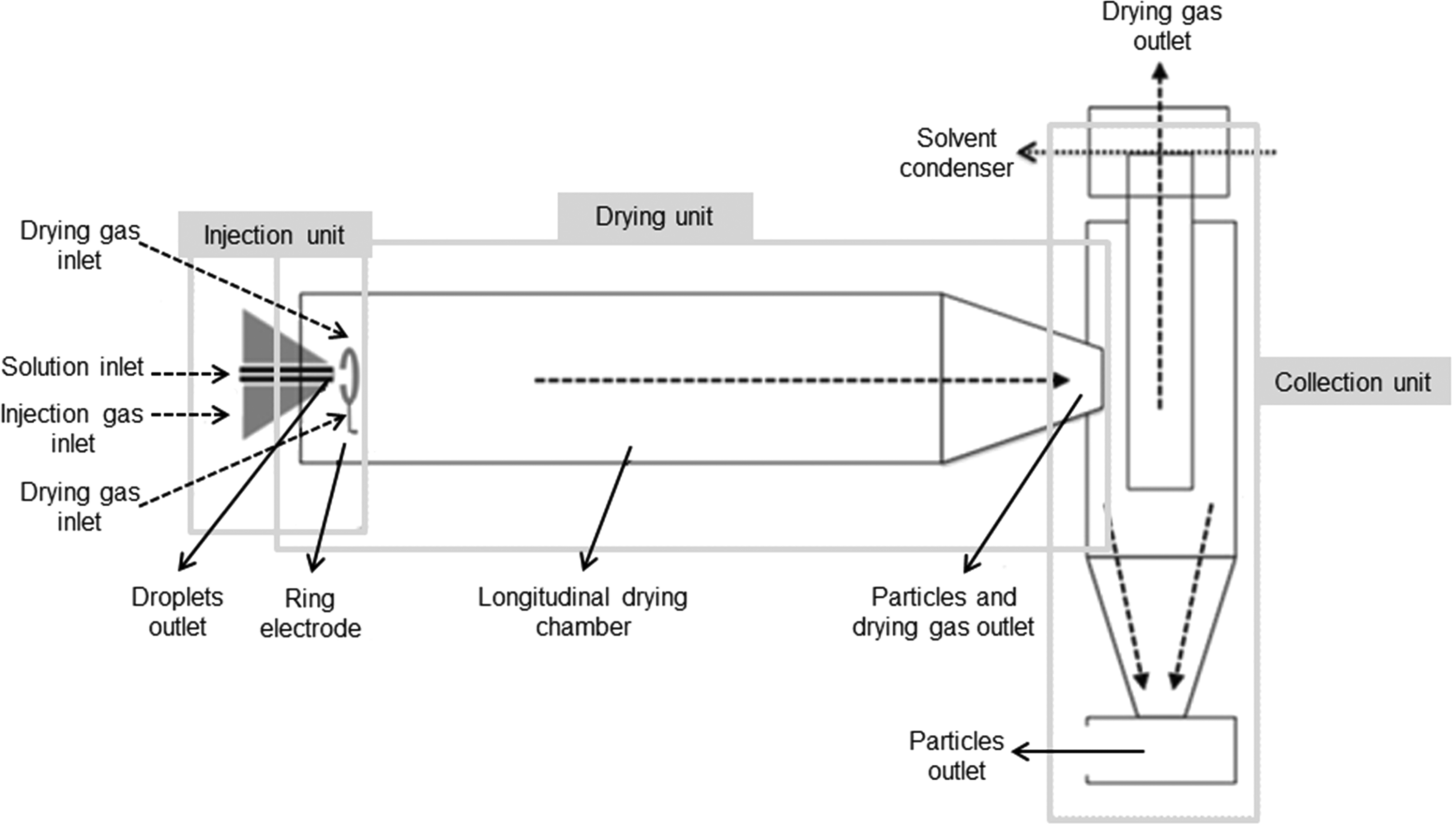
Scheme of the EAPG pilot installation used.

The five formulations shown in Table 1 were subjected to EAPG using the proprietary Capsultek pilot line from Bioinicia S.L. (Valencia, Spain). This pilot installation comprises a nebulizer that produces small droplets of the solutions, which are exposed after nebulization to an electric field to further dry and encapsulate by the created electrohydrodynamic forces, a drying chamber, and a cyclonic collector, as described elsewhere.^29^ A schematic diagram of the tool used is seen in Figure 1. The application of the electric field to the droplets exiting the nebulizer also helps to control the size of the resultant solution droplets as they fly toward the collecting unit. The experiments were optimally performed at controlled ambient conditions: 22 °C and 20% relative humidity (RH), a solution flow rate of 4 mL/min, voltage of 10 kV, and an air flow rate of 10 L/min. As a result, valsartan nanoparticles well dispersed and distributed within the excipient microparticles were obtained. The role of the polymeric excipients was to keep nanoparticles in a nonagglomerated state and at the same time to facilitate the handling. Drug nanoparticle size was controlled with process and solution parameters. Regarding throughput, the pilot unit used can produce around 6 g/h. However, the preindustrial equipment at Bioinicia S.L. facilities can produce in the range of kilos per hour. Since the EAPG process operates continuously, scaling of the technology to any volume is totally feasible.

### 2.4 Drug Content Determination

Valsartan containing microparticles (equivalent to 4 mg of valsartan) was dissolved in 100 mL of a phosphate-buffered solution (pH of 4.5) by magnetic stirring overnight. The solutions were analyzed spectrophotometrically at 250 nm (Dinko Instruments, Barcelona, Spain). The experiments were performed in triplicate. The analytical tests confirmed the presence of the drug in the expected quantities, confirming that no drug losses occurred during processing.

### 2.5 Electron Microscopy

Scanning electron microscopy was used to analyze the morphology of the valsartan microparticles in a Hitachi S-4800 FE-SEM (Hitachi High Technologies Corp., Tokyo, Japan) with an electron beam acceleration of 10 kV. The samples were coated with gold/palladium before SEM analysis. Particle size was measured using Image J Launcher v1.41 (National Institutes of Health, Bethesda) and the diameters presented were based on measurements from a minimum of 100 particles.

A transmission electron microscope (TEM) JEM-1010 (JEOL Ltd., Tokyo, Japan) was used to study the internal morphology of the microparticles. Samples were included in LR-White resin and the blend was polymerized at 60 °C for 48 h. An ultramicrotome Ultracut Leica EM UC6 (Leica Microsystems, Wetzlar, Germany) was used to cut ultrathin sections that were deposited over the TEM grid prior to TEM observation.

### 2.6 Particle-Size Distribution

The average particle-size distribution of the valsartan nanoparticles released from within the microparticles was also tested by photon correlation spectroscopy (Zetasizer Nano-ZS, Malvern Instruments Ltd., Malvern, U.K.). The microparticles were dissolved in a phosphate-buffered solution (pH = 6.8) at room temperature and at a concentration of 40 mg/L, with slow magnetic stirring for 30 s. A total of 1 mL of the previous solution was sampled and analyzed. Overall, 30 s was selected as the stirring time since in this time the dissolution of the polymeric matrix occurred, releasing the valsartan nanoparticles, without promoting the dissolution of said valsartan nanoparticles. The analysis was performed at 25 °C, in triplicate.

### 2.7 Differential Scanning Calorimetry (DSC)

A DSC-8000 from PerkinElmer Inc. (Waltham, MA), equipped with a cooling accessory Intracooler 2 also from PerkinElmer, Inc. was used to study the thermal transitions. A heating program was applied from 35 to 200 °C, at 10 °C/min, with a nitrogen flow rate of 20 mL/min. The sample weight was around 3 mg, while an empty aluminum pan was used as a reference. Indium was used to calibrate the DSC. All analyses were performed, at least, in duplicate. Pyris Manager software (PerkinElmer Inc., Waltham, MA) was used to analyze the thermograms.

### 2.8 Wide-Angle X-ray Scattering (WAXS)

A Bruker AXS D4 Endeavor diffractometer (Bruker, Ettlingen, Germany) was used to perform the wide-angle X-ray scattering measurements. The samples were scanned at scattering angles (2Θ) between 5 and 30°, at room temperature, in a reflection mode, using incident Cu Kα radiation (Cu Kα = 1.54 Å), the generator being set up at 40 kV and 40 mA.

### 2.9 Attenuated Total Reflection-Fourier Transform Infrared Spectroscopy (ATR-FTIR)

A Bruker Tensor 37 FT-IR spectrometer (Bruker, Ettlingen, Germany) coupled with the ATR sampling accessory Golden Gate (Specac Ltd., Orpington, U.K.) was used to obtain the ATR-FTIR spectra of approximately 50 mg of particles. All spectra were recorded between 4000 and 600 cm^–1^, with a resolution of 4 cm^–1^, and averaging 10 scans. Measurements were performed in triplicate. The software OPUS 4.0 (Bruker, Ettlingen, Germany) was used to analyze the spectral data.

### 2.10 In Vitro Dissolution Rate Test

Dissolution rate profiles of commercial valsartan and the here-obtained nanostructured microparticles were measured in a phosphate-buffered solution at pH 4.5 since valsartan is poorly soluble in acidic (pH ≤ 5) and nonbuffered solutions.^38^ A United States Pharmacopeia method II dissolution tester (Dissolution System 2100 C, Distek Inc., North Brunswick, NJ) was used for the *in vitro* dissolution testing. The dissolution test was performed at 37.0 ± 0.5 °C. Valsartan samples with an equivalent amount of 40 mg valsartan were located in powder form inside dissolution vessels containing 900 mL of a phosphate-buffered solution at pH of 4.5. The paddle rotation was 50 rpm. Aliquots (5 mL) were sampled during 40 min and filtered through 0.45 μm poly(tetrafluoroethylene) (PTFE) syringe filters. At each time point, an equal volume of a dissolution medium was added to the dissolution vessel. A UV/vis spectrophotometer (UV4000, Dinko Instruments, Barcelona, Spain) was used to determine the valsartan concentration at a detection wavelength of 250 nm. The analysis was performed in triplicate and the results are presented as the mean ± standard deviation.

### 2.11 Caco-2 Cell Model for In Vitro Transport Study

Caco-2 cells, a human colorectal adenocarcinoma cell line, are broadly used to evaluate the drug absorption *in vitro*.^39,40^ Briefly, cells were grown in the EMEM complete medium supplemented with 20% heat-inactivated FBS, penicillin-G (100 U/mL), streptomycin (100 μg/mL), and amphotericin B (0.25 μg/mL) in a humidified incubator with 5% CO_2_ and a 95% air atmosphere at 37 °C. Caco-2 cells with a passage number from 20 to 25 were seeded at a density of 1 × 10^5^ cells/well, on polycarbonate inserts in 12 Transwell plates (12 mm diameter, 0.4 μm mash). Experiments were performed after 18–21 days postconfluence, a necessary period to differentiate spontaneously into a polarized monolayer with microvilli and tight junctions.^41,42^ Every day, monolayer integrity was checked by measuring the transepithelial electrical resistance (TEER) with a Millicell-ERS-2 voltohmmeter (Merck-Millipore, Darmstadt, Germany). All experiments were performed with TEER values >300 Ω·cm^2^ in HBSS (donor and receptor compartment). A concentration of 250 μg/mL of VLS, valsartan particles in HPMC without Span 20 (VEH), HPMC with Span 20 (VEHS), lactose monohydrate without Span 20 (VEL), and lactose monohydrate with Span 20 (VELS) samples in HBSS was used for this analysis. Samples were collected at different time points: 0, 15, 30, and 60 min. The collected samples were analyzed to quantify valsartan concentration using the liquid chromatography with tandem mass spectrometry (LC-MS/MS) method. The apparent permeability coefficient (*P*_app_) from the donor part to the receptor part was estimated according to eq 1(40)
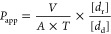
1where *V* is the volume (mL) of the receptor compartment, *A* is the membrane area (cm^2^), *T* is the time (s), [*d*_r_] is the concentration of the drug in the receptor compartment (ng/mL), [*d*_d_] is the concentration of the drug in the donor compartment (ng/mL), and the apparent permeability coefficient is expressed in cm/s.

### 2.12 Pharmacokinetic Study

Fifteen male Sprague Dawley rats (300 ± 20 g body weight) were randomly divided into three groups of five rats each. Rats were kept under standard laboratory conditions at 25 ± 0.2 °C, 55 ± 5% RH, and 12 h light schedule. Each animal received a single gelatin capsule filled with commercial valsartan, VEH and VEHS, the valsartan dose being 10 mg/kg. After oral administration, blood samples (approximately 0.25 mL) were collected in heparinized tubes from the tail vein catheter at specified time points during 24 h (0, 0.25, 0.5, 1, 2, 3, 6, and 24 h). Plasma samples for LC-MS/MS analytics were obtained by centrifugation at 13 000 rpm at 4 °C for 5 min. A total of 0.1 mL of plasma was mixed with 0.2 mL of acetonitrile for protein precipitation and vortexed for 3 min. All samples were centrifuged at 13 000 rpm at 4 °C for 10 min, and the supernatants were collected and stored in LC-MS/MS plates at −80 °C until the drug concentration was measured. The calibration curve was built up using the peak area in comparison with the nominal concentration in the range of 0.1–500 ng/mL (*r*^2^ = 0.994). Hydrochlorothiazide was used as an internal standard. The area under the curve (AUC_0→24h_), the maximum concentration of valsartan after oral administration (*C*_max_), and the time to reach the maximum concentration (*T*_max_) were determined from the experimental data. All experiments were carried out in compliance with the University of Valencia (Valencia, Spain) and were approved by the Ethics Committee of the same university (2019/VSC/PEA/0220).

### 2.13 Ultraperformance Liquid Chromatography Tandem Mass Spectrometry (UPLC-MS/MS)

The ACQUITY TQD (Waters, Milford, MA) equipped with a positive electrospray ionization was used for UPLC-MS/MS analysis. The valsartan was separated on an ACQUITY UPLC BEH C18 column (Waters, Milford, MA) (particle size 1.7 μm; 2.1 mm × 50 mm). The mobile phase consisted of acetonitrile and 0.1% formic acid in water (80:20), and was eluted at a flow rate of 0.4 mL/min.

The operating conditions of the mass spectrometer were optimized in the positive ionization multiple reaction monitoring (MRM) mode with an interchannel delay of 0.03 s. The transition of valsartan was 436 > 235.08 and 436 > 418.24. The capillary voltage was set at 3.5 kV and the source and desolvation temperature at 120 and 300 °C, respectively. The desolvation and cone gas flow rates were 680 and 25 L/h, respectively.

The mass spectrometer was equipped with a Z-spray electrospray ionization source and the samples were analyzed with the following conditions: capillary: 3.5 KV, extractor: 5 V, RF lens: 0.3 V, source temperature: 120 °C, desolvation temperature: 300 °C, cone gas: 25 L/h, and desolvation gas: 680 L/h. MS1 parameters were LM resolution, 13; HM resolution, 13; ion energy, 1. MS2 parameters were LM resolution, 13; HM resolution, 13; ion energy, 0.7; multiplier 650 V. Spectra were acquired in a positive ionization multiple reaction monitoring (MRM) mode with an interchannel delay of 0.03 s and the transition of valsartan was 436 > 235.08 and 436 > 418.24.

### 2.14 Statistical Analysis

Statistical analysis of the *in vitro* drug permeability and of *in vivo* pharmacokinetics was performed by one-way analysis of variance (ANOVA), and Tukey’s multiple comparison tests were used as the post-hoc test. *p* ≤ 0.05 was considered statistically significant. The software used was Statgraphics Centurion XVI (StatPoint Inc., Warrenton, VA).

## 3 Results and Discussion

### 3.1 Microparticle Morphology

**Figure 2:**
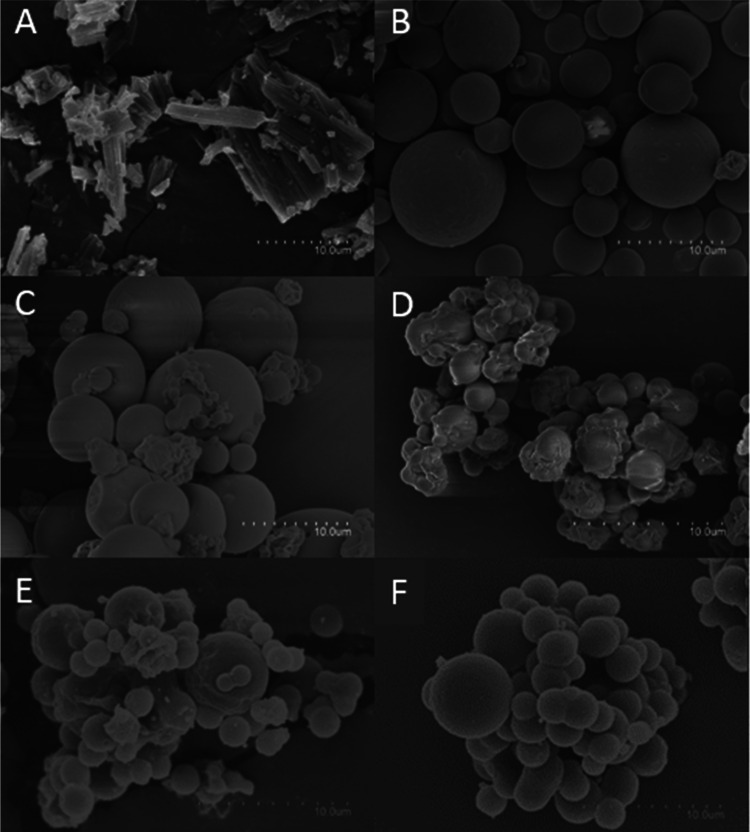
SEM images of (A) commercial valsartan, (B) VLS, (C) VEHS, (D) VEH, (E) VELS, and (F) VEL. The scale bar in all images was 10 μm.

Figure 2 shows a comparison of the morphology of the as-received commercial valsartan microparticles (Figure 2A) and of the various formulations of valsartan dissolved and processed by the EAPG technology (Figure 2B–G). While the commercial valsartan has a crystalline acicular form with broad particle size (Figure 2A), spherical microparticles with a smooth surface and size of around 4.8 μm were obtained by EAPG (Figure 2B). Formulations produced with HPMC (Figure 2C,D) showed a rough surface and mean size around 4.5 μm, whereas microparticles created with lactose monohydrate showed a mean size around 3 μm and a smooth surface with a certain degree of agglomeration, probably due to the lactose monohydrate hygroscopicity (Figure 2E,F). The presence of the surfactant did not create any significant differences regarding the microparticles’ external morphology between samples.

The appearance of the microparticles produced with HPMC was similar to that observed by other authors using other techniques. Thus, Yan et al. produced solid dispersions of valsartan in HPMC *via* spray-drying, with a drug loading between 50 and 80%, and obtained highly agglomerated particles, with a relatively rough surface and an average particle size of 4 μm. These authors attributed the rough surface to the attachment of the polymer and the surfactant to the surface of the drug.^20^

### 3.2 Morphology of Valsartan within the Microparticles

**Figure 3:**
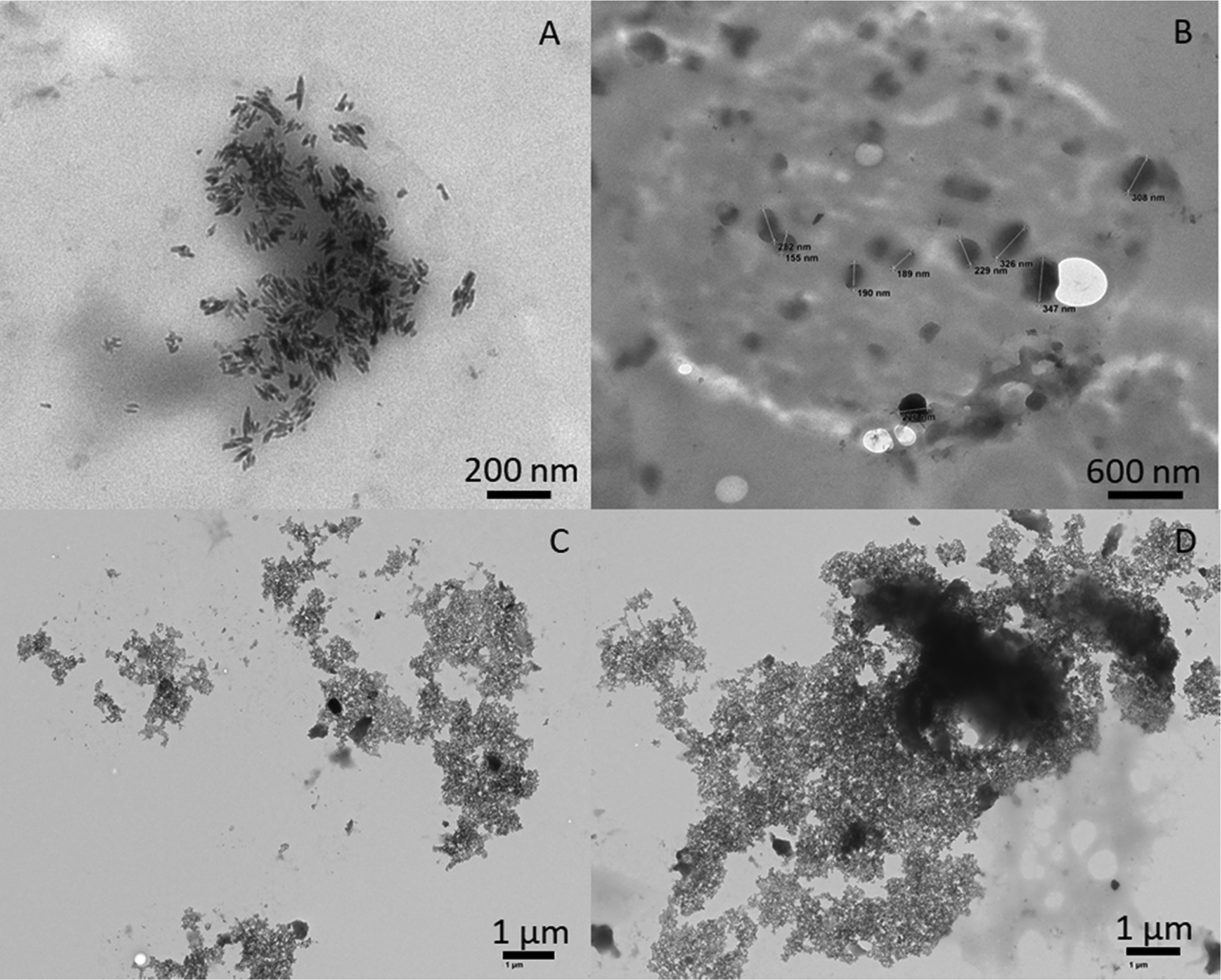
TEM image of the valsartan particles in (A) HPMC with Span 20 (VEHS), (B) HPMC without Span 20 (VEH), (C) lactose monohydrate with Span 20 (VELS), and (D) lactose monohydrate without Span 20 (VEL).

The size of the drug alongside the crystalline morphology and content inside the microparticles is thought to play a key role in its solubility and bioavailability. The use of an emulsion, which acts as a template, is an alternative to control the drug particle size inside the microparticles, which affects the solubilization rate. The size of valsartan inside of the microparticles was characterized by photon correlation spectroscopy and TEM. Sizes below micron were obtained for all of the formulations, being larger for the particles without a surfactant due to the larger emulsion droplet size obtained without the surfactant. For instance, satisfactory results obtained *via* photon correlation spectroscopy indicated that valsartan mean size for the formulation with HPMC and Span 20 (VEHS) was 147 nm (PDI of 0.401), whereas the valsartan mean size was 264 nm (PDI of 0.442) for the formulation without the surfactant (VEH). In the case of lactose monohydrate, the differences were more significant, with a size of 408 nm (PDI of 0.379) for the formulations with Span 20 (VELS) and 655 nm (PDI of 0.348) for the formulations without Span 20 (VEL). These results were corroborated by TEM (see Figure 3). Figure 3 indicates that in valsartan in HPMC with (VEHS) and without Span 20 (VEH), the API particle size was found to be around 150 and 300 nm, respectively. Samples prepared with lactose monohydrate were more difficult to cut with the ultramicrotome due to the fragility of the internal structure of the lactose monohydrate; however, images obtained by TEM corroborated the previous results obtained by photon correlation spectroscopy, the valsartan particle size being around 400 and 650 nm for the formulations with and without Span 20, respectively. Ma et al. produced micro- and nanoparticles of valsartan by antisolvent precipitation followed by spray-drying, obtaining particles between 2 and 60 μm in the case of valsartan microsuspension and between 30 and 117 nm in the case of valsartan nanosuspensions.^15^ However, this technique required the presence of Aerosil 200 to avoid agglomeration, as well as the use of high temperature to dry the particle suspension.

### 3.3 Differential Scanning Calorimetric Analysis

**Figure 4:**
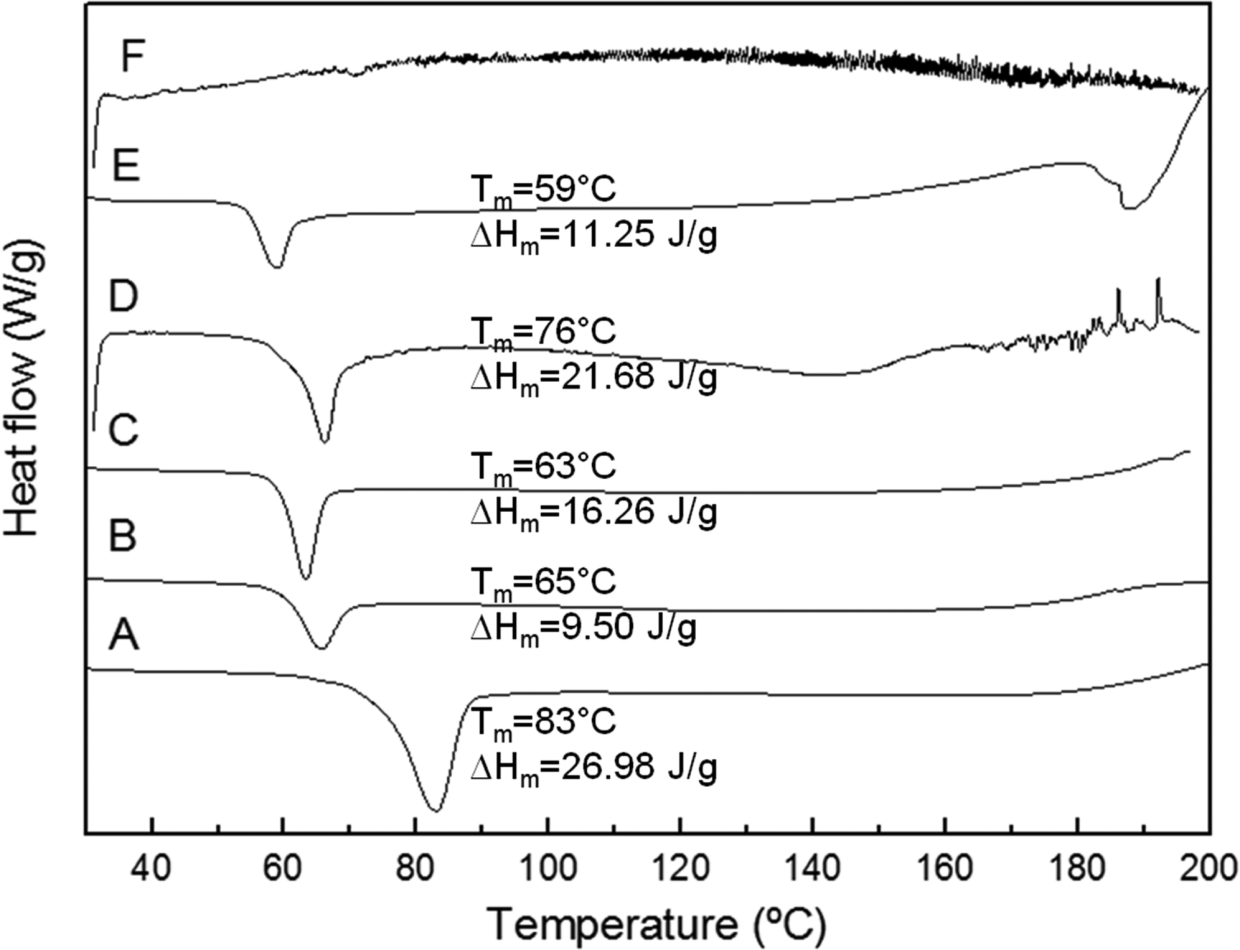
DSC thermograms of commercial valsartan (A), VLS (B), VEHS (C), VEH (D), VELS (E), and VEL (F).

To investigate the crystallinity of the valsartan in the microparticles, DSC measurements were performed. The analysis of the melting peak of the drug in the thermograms was used as an indicator of its crystalline phase in the sample. Figure 4 shows the thermograms of the various samples produced. The commercial valsartan thermogram exhibited a single sharp endothermic melting peak at 83 °C with an enthalpy of 26.98 J/g. Similar results were reported by some authors.^43^ However, other authors have reported a melting peak of valsartan at 112 °C,^44^ a feature that was not observed in the commercial sample reported in this study. Regarding the valsartan sample processed by EAPG, it showed a melting point drop of 18 °C and a melting enthalpy reduction of 17.48 J/g, suggesting that a more defective less crystalline morphology was achieved by the process. The same behavior was observed for the samples prepared with HPMC and lactose monohydrate, the melting temperature being reduced by more than 6 °C. The enthalpy also decreased, compared to the neat drug, in at least by 5 J/g, the samples with lactose monohydrate being the ones that showed the maximum reduction, with a melting enthalpy lower than 12 J/g.

### 3.4 Wide-Angle X-ray Scattering (WAXS)

**Figure 5:**
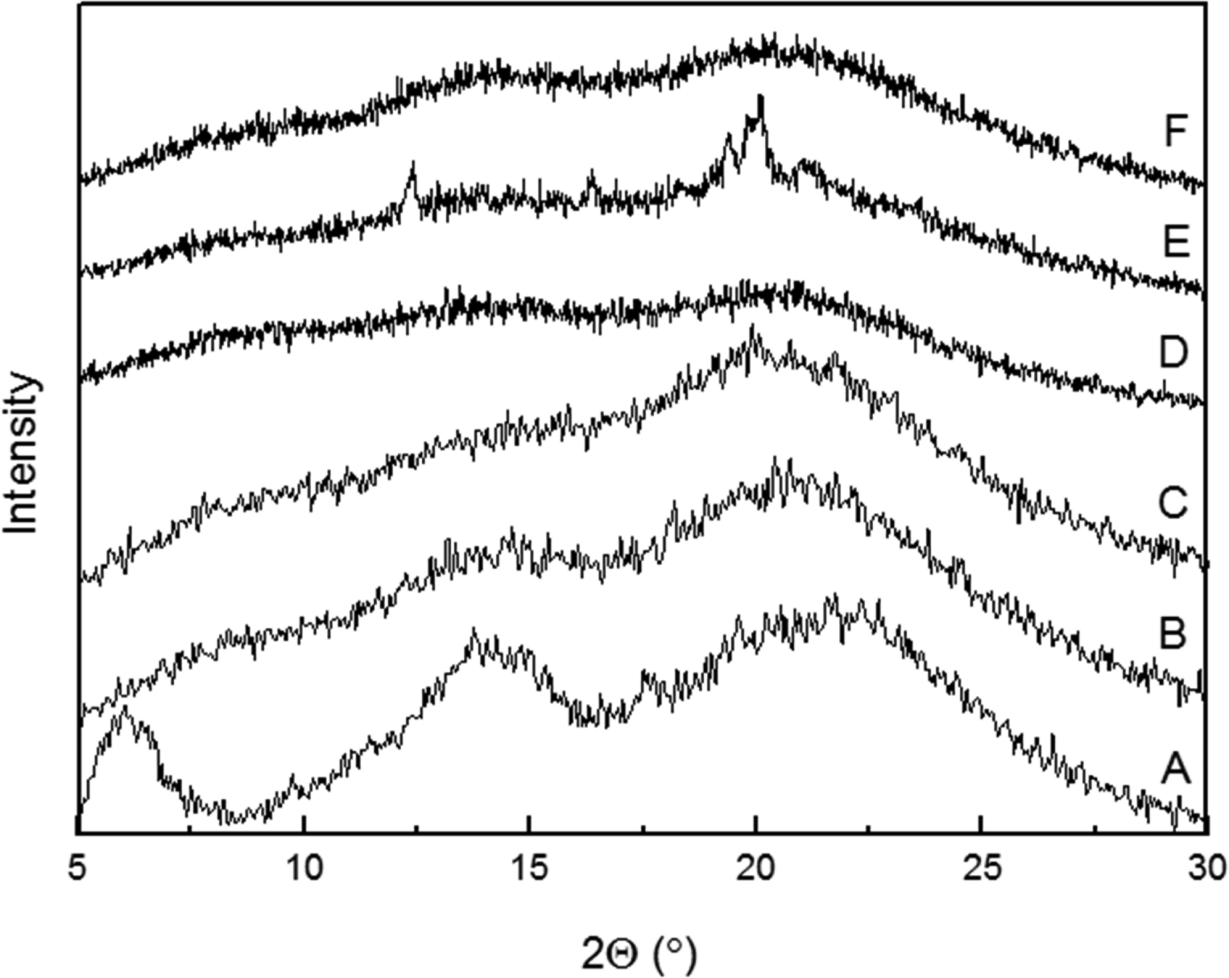
Wide-angle X-ray scattering diffractogram of (A) commercial valsartan, (B) VLS, (C) VEHS, (D) VEH, (E) VELS, and (F) VEL.

Wide-angle X-ray scattering analysis was also used to assess the crystallinity of the materials. As mentioned above, any reduction in crystallinity and/or particle size of the drug is expected to lead to an enhanced dissolution rate.^45^ The diffraction patterns of the studied valsartan formulations are shown in Figure 5. From Figure 5, the diffraction patterns of the pure valsartan showed broad peaks, suggesting that the crystallinity of the commercial product is already low (Figure 5A). The patterns of the valsartan samples processed by EAPG showed even weaker peaks, in agreement with the DSC data, suggesting that a higher degree of amorphicity of the API is obtained when processed through EAPG (Figure 5B–F).

### 3.5 Attenuated Total Reflection-Fourier Transform Infrared Spectroscopy (ATR-FTIR)

**Figure 6:**
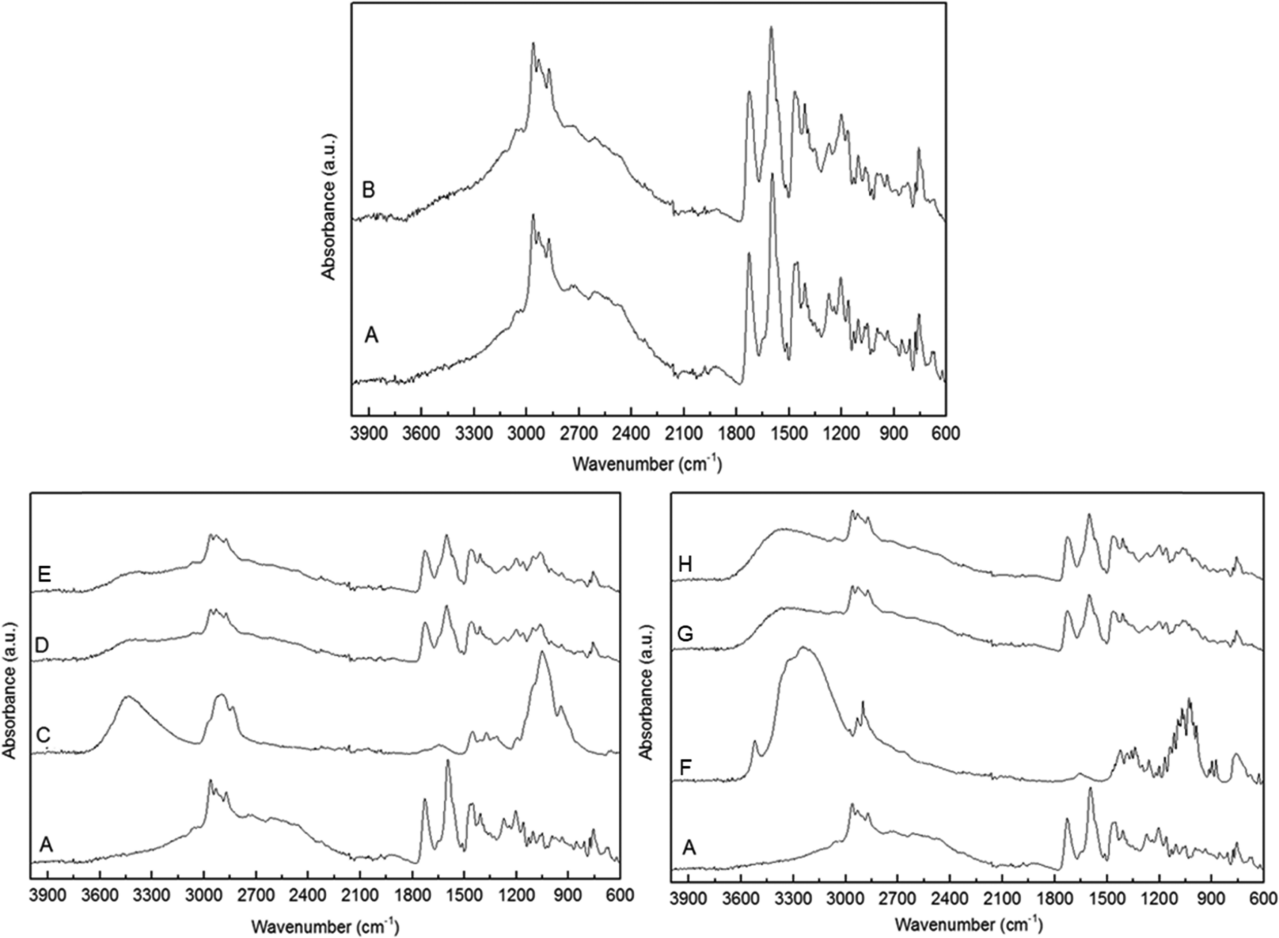
ATR-FTIR spectra of commercial valsartan (A), VLS (B), HPMC (C), VEHS (D), VEH (E), lactose monohydrate (F), VELS (G), and VEL (H).

The ATR-FTIR spectra of the raw materials and the prepared formulations are shown in Figure 6. ATR-FTIR spectroscopy was used as an analytical tool for the detection of potential interactions and changes in the molecular order between valsartan and the excipients used. The ATR-FTIR spectra of commercial valsartan in Figure 6A show a broad band at 3300 cm^–1^ arising from stretching vibrational modes of O–H and N–H functional groups. The band at 2962 cm^–1^ is assigned to the C–H group stretching vibration of the aromatic ring. The peak at 1728 cm^–1^ reveals the presence of a carboxylic functional group. The characteristic peak at 1597 cm^–1^ is ascribed to the stretching of a N=C=O bond in the amide functional group present in the drug structure, and the presence of a band at 1513 cm^–1^ is assigned to the N=N bond. The band at 1455 and 1466 cm^–1^ is assigned to the aromatic C=C vibrations, the peak at 1331 cm^–1^ arises from the C=N bond, the peak at 1051 cm^–1^ is ascribed to the C–N bond, while the bands in the range of 1207–1025 cm^–1^ are ascribed to the presence of a tetrazole ring (CN_4_). These vibrational assignments are based on the previous literature.^46^ The ATR-FTIR spectrum of the VLS sample in Figure 6B did not show any significant band shifts, but a broadening of some of the bands, most likely due to the lower degree of crystallinity suggested by the DSC and WAXS results.

Figure 6A,C,D,E compares the ATR-FTIR spectra of the samples prepared with HPMC and the raw materials. The HPMC spectrum (Figure 6C) is characterized by three main bands, corresponding to the very intense elongation of the O–H bond at 3446 cm^–1^, the band attributed to C–H elongation at 2935 cm^–1^, and the band at 1055 cm^–1^ assigned to the asymmetric elongation of the C–O–C of the bonds *O*-glycosidic β-(1-4).^47^ The ATR-FTIR spectra of the samples prepared with HPMC presented the characteristics bands of valsartan and HPMC, with also no significant band shifts, but with some of the bands undergoing broadening, the effect that suggests reduced crystallinity.

Figure 6A,F,G,H shows the ATR-FTIR spectra of the formulations prepared with lactose monohydrate and of the raw materials used. The ATR-FTIR spectra of lactose monohydrate (Figure 6F) presented a sharp medium band at 3528 cm^–1^ arising from the vibration of the O–H band of sorbed water.^48^ Samples with lactose monohydrate (Figure 6G,H) showed no unambiguously discernible interactions between the drug and the excipients but showed a clear band broadening assigned as above to a decrease in the molecular order for the API.

### 3.6 Dissolution Rate Tests

**Figure 7:**
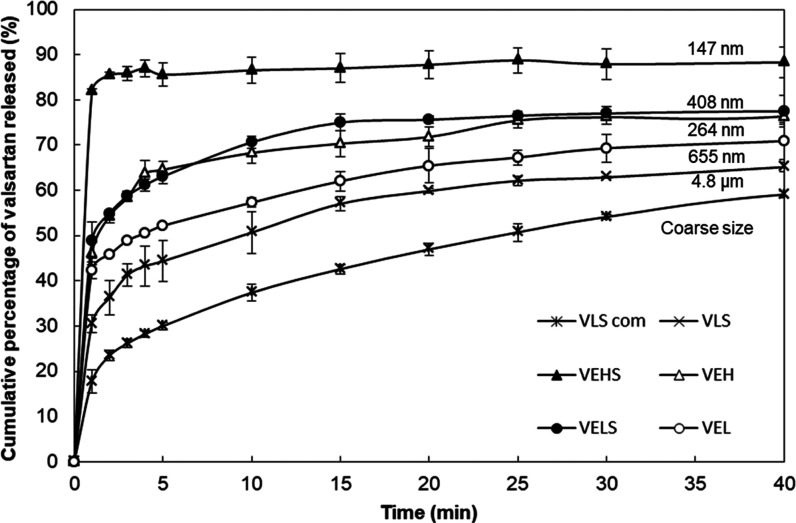
Comparison of the dissolution profiles of valsartan samples in the powder form, where VLS com is the commercial valsartan, VLS is the valsartan processed by EAPG, VEHS is the valsartan in HPMC with Span 20, VEH is the valsartan in HPMC without Span 20, VELS is the valsartan in lactose monohydrate with Span 20, and VEL is the valsartan in lactose monohydrate without Span 20. Valsartan particle size was included in the figure for ease of comparison. The deviation among different experiments was ≤4%.

The dissolution profiles of commercial valsartan, valsartan processed by EAPG, and here-obtained formulations in an enzyme-free phosphate-buffered solution (pH 4.5) are shown in Figure 7. The deviation between experiments was less than 4%, and consequently, at several time points, the error bars remained behind the marker. The observed dissolution rate of commercial valsartan was 40% in the first 10 min, whereas the valsartan processed with the EAPG technology reached 51% of cumulative release in the same time. Regarding the here-obtained formulations with excipients, it seems that valsartan was released in a biphasical model with an initial fast dissolution rate due to a burst release, followed by a slower one. The sample prepared with HPMC and Span 20 (VEHS) reached a cumulative release of around 80% in the first 10 min; however, without Span 20 (VEH), the cumulative release in the same time decreased to 60%. The sample with lactose monohydrate and Span 20 (VELS) showed a cumulative release around 70% in the first 10 min, which decreased to 57% without Span 20 (VEL).

According to Figure 7, the enhancement in dissolution rate is primarily related to the drug particle size per the same excipient. However, when the different microparticles are compared, there seems to be also an effect arising from the presence of the surfactant. Thus, for the HPMC microparticles containing API sizes of 264 nm, the dissolution rate is similar to the lactose monohydrate microparticles with Span 20 containing API sizes of 408 nm. This effect could be ascribed to a potential increase in wettability of the highly insoluble API induced by the surfactant. Other authors have also discussed enhanced dissolution rates when preparing solid dispersions *via* spray-drying or freeze-drying. Thus, Yan et al. prepared valsartan solid dispersions in HPMC with sodium lauryl sulfate (SLS) as a surfactant *via* spray-drying with a valsartan loading around 80%, and obtained a cumulative valsartan release of less than 2% at pH = 4 and 70% at pH = 6.8.^20^ Xu et al. reached a cumulative release of valsartan of 90% in the first 20 min at pH = 6.8 by preparing valsartan solid dispersions in HPMC with poloxamer 188 as a surfactant *via* freeze-drying, with valsartan loadings around 23%. However, without the surfactant, the cumulative release of valsartan only reached 30% in the first 20 min.^43^ Ma et al. compared the dissolution rate of micro- and nanoparticles of valsartan with poloxamer 407 and Aerosil 200 prepared by antisolvent precipitation followed by spray-drying, obtaining for both sizes a valsartan release of 90% in the first 10 min at pH = 4.^15^ However, this method presents the disadvantages of using of high temperature, which could affect drug stability, and a high concentration of Aerosil 200 to avoid agglomeration.

### 3.7 Caco-2 Cell Line Permeability Study

**Figure 8:**
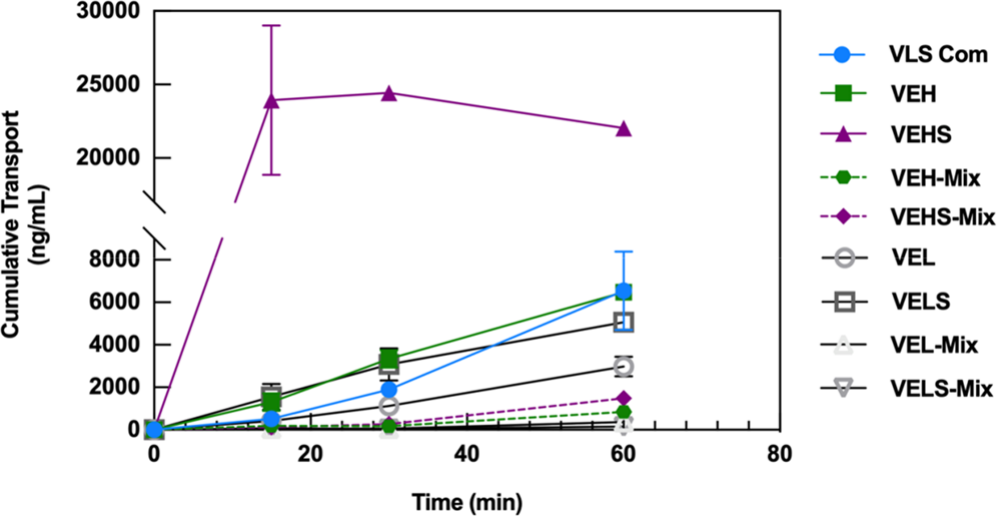
Caco-2 permeability profile of commercial drug (VLS Com), valsartan in HPMC (VEH), HPMC with Span 20 (VEHS), lactose monohydrate (VEL), and lactose monohydrate with Span 20 (VELS). The physical mixture of valsartan with HPMC (VEH-Mix), HPMC with Span 20 (VEHS-Mix), lactose monohydrate (VEL-Mix), and lactose monohydrate with Span 20 (VELS-Mix) were used as controls. The treatment concentration was of 250 μg/mL (mean ± SD, *n* = *3*).

The Caco-2 cell monolayer was used as an *in vitro* model of the intestinal mucosa to assess the gastrointestinal permeability of commercial valsartan (VLS) and the prepared valsartan samples. This study was performed with the formulations that provided a faster dissolution profile in Figure 7. As shown in Figure 8, the samples prepared with HPMC and Span 20 (VEHS) showed the highest increase in the permeability rate; after 15 min, the drug transport across the monolayer resulted in more than 10-fold increase compared to the commercial form of the drug. In Figure 8, the error bars at several time points were so small that remained behind the marker. This increase in the permeability rate is primarily ascribed to the nanonization of the API but also to the presence of Span 20 in the VEHS formulation. The effect of surfactants as permeability enhancers in Caco-2 experimental models is well documented.^49,50^ In any case, the results using physical mixtures of the various formulations confirmed that the ability to permeate is not solely caused by the presence of the surfactant. No significant differences were observed among the other prepared samples compared to the commercial drug, even though a faster crossing capacity through the Caco-2 monolayer for VEH and VELS during the first 15–30 min was observed.

The apparent permeability values (*P*_app_) obtained using the Transwell model for all previous conditions for commercial valsartan and the here-prepared samples are gathered in Table 2. These results corroborate the significant enhancement in the Caco-2 cells monolayer permeability of the valsartan samples prepared with HPMC and Span 20 compared to the pure commercial drug. The apparent permeability of the commercial valsartan was 6.60 ± 1.86 × 10^–6^ cm/s, which is higher than the one previously reported by Lin et al. of 0.4 × 10^–6^ cm/s.^51^ However, the apparent permeability of the VEHS formulation was 4-fold faster than the pure drug (22.26 ± 0.39 × 10^–6^ cm/s). Improvements of apparent permeability of valsartan samples through the Caco-2 cell monolayer had already been reported by Nekkanti et al. by the development of proliposomal formulations but without reaching 10 × 10^–6^ cm/s.^13^

**Table 2:** Apparent Permeability (*P*_app_) Evaluation of Commercial Valsartan and Here-Prepared Samples with HPMC (with and without Span 20) and Lactose Monohydrate (with and without Span 20) Using the Transwell Model with Caco-2 Cell Line

**formulation**	***P***_**app**_**for Caco-2 permeability** (× 10^**–6**^ **cm/s)**
VLS commercial	6.60 ± 1.86
VEH	6.69 ± 0.43
VEHS	22.26 ± 0.39
VEL	3.00 ± 0.03
VELS	5.12 ± 2.63
VEH-Mix	0.85 ± 0.21
VEHS-Mix	1.51 ± 0.83
VEL-Mix	0.37 ± 0.26
VELS-Mix	0.15 ± 0.04

### 3.8 Pharmacokinetic Study in Rats

**Figure 9:**
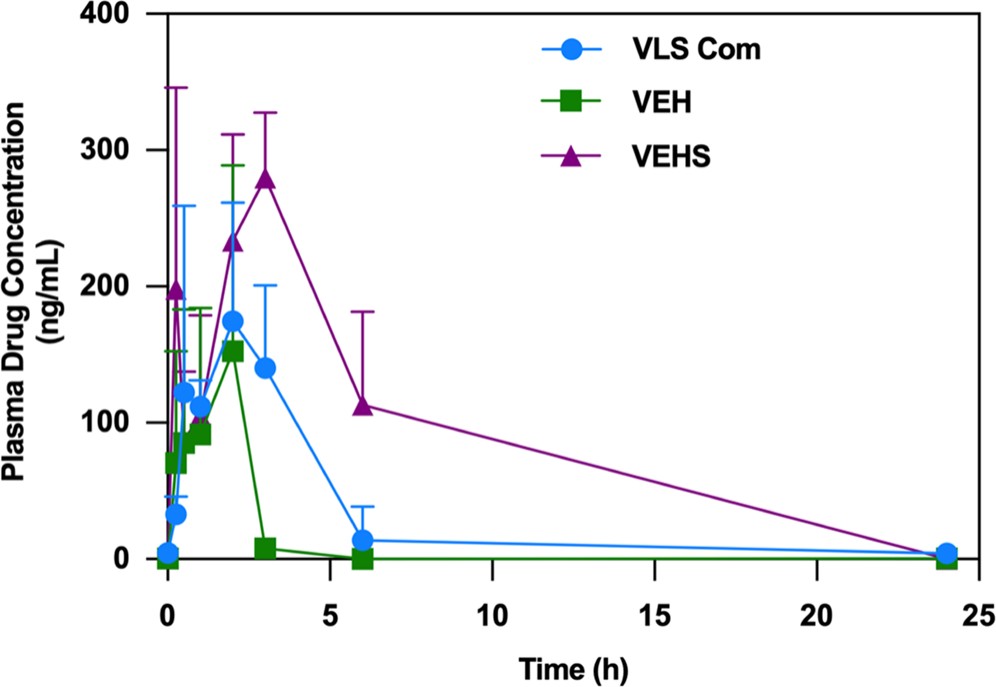
Mean plasma concentration of valsartan formulations (VEH and VEHS) and commercial drug following oral administration (mean ± SD, *n* = 5).

The enhanced permeability of the here-obtained formulations on the *in vitro* model with Caco-2 cells often translates into an improved *in vivo* intestinal absorption after oral administration. Only the formulations that provided the best results in permeability through Caco-2 cells, i.e., VEH and VEHS, were assayed and compared to the commercial valsartan formulation. The obtained mean plasma concentration versus time and the pharmacokinetic parameters (*T*_max_, *C*_max_, and AUC_0→24_) are gathered in Figure 9 and Table 3, respectively. The results of the VEHS formulation showed an increase in *C*_max_ (279.80 ± 47.80 ng/mL) compared to the commercial drug (174.70 ± 168.96 ng/mL). The 1 h delay to reach the maximum concentration may be due to the time necessary to release the drug from the microparticles within the intestine. The systemic exposure (AUC_0→24_) of VEHS was 3-fold higher in comparison to the pure commercial valsartan. Finally, a similar systemic behavior was seen between the commercial drug and VEH with regard to *T*_max_ and *C*_max_, albeit the AUC_0→24_ of the commercial valsartan is about 2-fold higher than for VEH.

**Table 3:** Pharmacokinetic Parameters of Commercial Valsartan, VEH, and VEHS Formulations Following Oral Administration in Rats (Mean ± SD, *n* = 5)

**PK Parameters**	**VLS Com**	**VEH**	**VEHS**
*T*_max_ (h)	2.00	2.00	3.00
*C*_max_ (ng/mL)	174.70 ± 168.96	152.50 ± 136.21	279.80 ± 47.80
AUC_0→24_ (ng × h/ mL)	778.50 ± 262.90	283.40 ± 113.80	2145.00 ± 631.50

The low permeability of valsartan has led to multiple strategies to increase its bioavailability. Baek et al. produced valsartan redispersable emulsions by spray-drying using HPMC and poloxamer 407 as excipients, reporting AUC_0→24_ values around 10 μg × h/mL in rats after oral administration with a valsartan loading of 4%.^10^ Nekkanti et al. proposed the use of proliposomes to improve the intestinal absorption and they obtained a *C*_max_ amelioration, but with an AUC_0→24_ value lower than 700 ng × h/mL.^13^ Ma et al. compared the bioavailability of micro- and nanoparticles of valsartan produced by antisolvent precipitation followed by spray-drying in *in vivo* assays, confirming the bioavailability enhancement of the nanosized form.^15^ The good results achieved in this study provide a strong support for the potential use of the EAPG technology to improve the bioavailability of nano-within-micro valsartan formulations and potentially also of other BCS class II drugs.

## 4 Conclusions

This is the first study that deals with the controlled nanonization of a pharmaceutical compound by virtue of a continuous scalable hybrid nebulization/electrospraying technology. This study was aimed at the development of nanostructured valsartan highly dispersed and distributed within excipient microparticles to display an enhanced dissolution rate and bioavailability, *via* the high-throughput electrospraying assisted by pressurized gas (EAPG) room-temperature processing technology. The work demonstrated that it was possible to obtain controlled nanoscale drug size morphologies with a high API loading, i.e., 80%. As a result, it was possible to customize the dissolution rate and bioavailability of a model BCS class II drug, such as valsartan. The developed formulations attained *ca*. 4 μm microparticles with drug particle sizes within them spanning between 150 and 650 nm, which resulted in 1.3- and 2.5-fold faster dissolution rates, respectively. During the *in vitro* and *in vivo* tests, the sample formulated with HPMC and Span 20 showed 4-fold faster *in vitro* permeability and 3-fold higher systemic exposure than the commercial valsartan. The work also demonstrated that it is feasible to derive disaggregated nanonized pharmaceutical formulations, which are safe to handle and with reduced crystallinity.

Future work will deal with a more detailed pharmacokinetic study in larger animals and humans. Additionally, in view of the very promising results obtained with valsartan, other BCS class II and IV molecules are being currently investigated by this technology.
